# Intrapulmonary Shrapnel

**Published:** 1916-10

**Authors:** 


**Intrapulmonary Shrapnel.** Petit de la Visseon, Soc. Chir., Nov. 30, 1915, reports a case of repeated small haemoptyses for 7 months after a wound of the chest. Operation, with the assistance of radioscopy, consisted in opening the lung with the thermocautery, removal of the mass, weighing 20 grams and 6x1x1½ c.m., suture of the lung en masse. Three months later, perfect recovery. (Note: Visceral operations, of many kinds, inevitably produce occlusion of various tubules, by wholesale. We do not recollect any general study of the exact processes involved, by which serious results are prevented, nor of residual dangers from this mechanic factor. Such a study occurs to us as a valuable form of research in surgical pathology. Ed.)

